# Reconciling the pHe measurements of bioethanol: pH_abs_ measurements of buffered 50-50 wt% water-ethanol mixtures

**DOI:** 10.1016/j.acax.2022.100085

**Published:** 2022-06-22

**Authors:** L. Deleebeeck, A. Snedden, D. Stoica

**Affiliations:** a DFM A/S, Kogle Allé 5, 2970, Hørsholm, Denmark; b Laboratoire National de Métrologie et d’Essais (LNE), 1 Rue Gaston Boissier, 75015, Paris, France

**Keywords:** ${\text{pH}}_{\text{abs}}^{{\text{H}}_{2}\text{O}}$Unified pH scale, Differential potentiometry, Borate, Phosphate, Phthalate

## Abstract

Water-ethanol mixtures intended for specific purposes, such as bioethanol fuel, can be subject to national quality standards, including the measurement of pHe – a solvent-specific quantification of acidity. This work discusses the shortcomings of the use of pHe in these quality standards, including the lack of metrological traceability of pHe measurements made using combination pH electrodes calibrated using aqueous pH buffers. The feasibility of measuring the acidity of 50-50 wt% water-ethanol mixtures on a non-solvent-specific, unified pH scale, which is traceable to the conventional aqueous pH scale (${\text{pH}}_{\text{abs}}^{{\text{H}}_{2}\text{O}}$) is demonstrated. ${\text{pH}}_{\text{abs}}^{{\text{H}}_{2}\text{O}}$ measurements of buffered and un-buffered water-ethanol mixtures using two cell configurations, including the use of an ionic liquid salt bridge (ILSB), show good agreement. The cell configuration, consisting of a commercial glass (half-cell) electrode and a reference electrode incorporating an ILSB, can be readily adopted by measurement laboratories.

## Highlights

• Assessment of different scales used to express the acidity of water-ethanol mixtures.
• Application of the absolute pH concept on (un)buffered 50 wt% water-ethanol mixtures.
• Differential potentiometric measurement setups for measuring absolute pH.
• Double junction reference electrode with ionic liquid for absolute pH measurement.
• Comparability of acidity measurements in complex solutions.

## Graphical abstract

**Figure:**
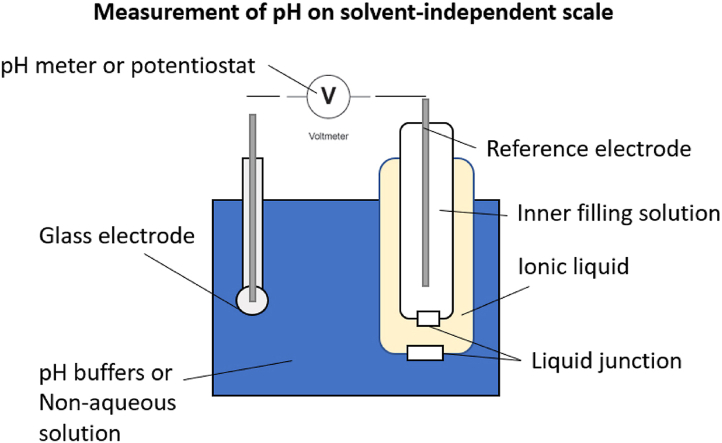

## 1 Introduction

Biofuels, including bioethanol, are expected to contribute towards the ‘greening’ of power utilization in the EU, and worldwide, especially in the transport industry. According to the Renewable Energy Sources Act of 2009, by 2050 the Danish government expects to reach 100% renewable energy in the energy and transport sectors [1]. Bioethanol is already included in the fuel mix in Brazil (e.g., E95), and various EU nations. With increased usage of bioethanol, its international trade is expected to become more significant. For example, one of the major producers of bioethanol from sugar cane is Brazil [2]; making Brazil well placed to become exporter of bioethanol. Trade in substances is often regulated by national or international quality standards, which include required measurands (quality metrics) which establish an agreed upon quality for the substance being traded. There exist several such standards for the use of bioethanol as an automotive fuel [3,4], including Brazil [5], USA [6], and the EU [7], and Japan [8]. The Brazilian and American standards require measurement of a parameter termed pHe – the pH of high purity denatured fuel ethanol – this measurand has been suggested as one that may be adopted into other national standards once a standard methodology is agreed upon [3,9].

The quantity pHe applies only to low water content bioethanol; where the solvent is composed primarily of ethanol, with the water content below a prescribed limit. It is worth noting that this prescribed limit currently differs between different national standards [3,4]. All ‘pure ethanol’ solutions, i.e., anhydrous ethanol and anhydrous bioethanol, include a certain amount of water. This is especially true after the ethanol solvent comes into contact with moist air, as it begins absorbing water vapor [10]. As such, it is essentially impossible to measure a sample that is 100% ethanol, all real solutions are water-ethanol mixtures.

The American standard (ASTM D6423-14 [6]) includes the method-defined means of measuring the pHe measurand. This method requires the use of a specific electrode, includes detailed measurement constraints, and suggests traceability is achieved through calibration of the specified electrode using aqueous buffer solutions. Reports on measurement of pHe in bioethanol originate with national metrology institutes (NMIs) in Brazil [[11], [12], [13], [14], [15]], the EU [[16], [17], [18]], and a national research center in the USA [19]. The significant issues with the ASTM method of measuring pHe have been discussed in Brown et al. [17]. Issues arising from the use of various glass-electrodes in water-ethanol mixtures has been discussed by Ref. [11], while differences between ASTM [6] and various other methods (including EN 15490:2007–10 [7]) are highlighted in Ref. [18].

From a metrological point of view, pHe is faced with some drawbacks. Firstly, there are some issues regarding the concept and the definition of pHe itself. In ethanol, or its mixtures with water, the measured pH is analogous to, but not interchangeable with, the conventional (aqueous) pH scale [20]. The measured pHe values are impossible to compared in terms of their effect on proton activity as each water-ethanol mixture, considered as a continuous solvent, has its own pH scale whose width depends on the solvent autoprotolysis constant [21,22].

From a practical point of view, the method of measuring pH in solvents other than pure water is based on differential potentiometry, using glass electrodes to sense protons (H^+^) and a reference electrode immersed in a filling solution, typically 3 M KCl (aq). The current practices follow the existing International Union of Pure and Applied Chemistry (IUPAC) methodology, recommended for low ionic strength aqueous solutions (ionic strength below 0.1 mol kg^−1^). However, in low water content solvent mixtures, and generally in non-aqueous organic solvents, its applicability is restricted (often to the point of being useless) primarily due to:

(i) Poor performance of the commonly used glass electrodes in non-aqueous media. One of the major issues with the use of aqueous buffer calibrated glass electrodes, including those specifically marketed for the measurement of ethanol [11,14,15,23], is the unknown magnitude of the liquid junction potential (LJP) between the pH electrodes' reference electrode filling solution and the non-aqueous solution under test. Further, glass electrodes behave differently when in contact with aqueous solutions vs. mixed water-ethanol solutions. This is related to changes in the layer of hydration (hydrogel) on the surface of the glass electrode proton sensing membrane [17,24].
(ii) Traceability of routine measurements on a pHe scale should be achievable in the same manner as the conventional pH scale i.e., through the use of metrologically traceable buffered ethanol certified reference materials (CRMs) [13]. The use of solvent mixture-specific pH scales would require a very high number of primary pH realizations (different non-aqueous solvents, solvent ratios, buffer salts, temperatures [25]) and the production of a large number of secondary pH buffer CRMs. The sheer number of CRMs required is impossible for NMIs to fulfill.
(iii) Because of solubility problems, the availability of suitable standard buffers in pure ethanol is limited. Instead, attempts have been made to characterize a series of buffer solutions prepared in various water-ethanol mixtures. Assignment of standard pH values has been done based on measurements with cells without transference, called Harned cells, by transposing the procedure carried out for classical aqueous buffer solutions, [20,26]. A limited series of buffered water-ethanol mixtures, at various temperatures, have been characterized in this manner [[27], [28], [29], [30], [31]].

However, a step forward in the measurement of acidity in any media has been proposed [32] as an absolute pH scale, noted pH_abs_, which is inter-convertible with the conventional pH scale [22]. pH_abs_ expresses a direct measure of the hydrogen ion chemical potential in any given medium and relies on a universal, solvent–independent standard state, taken as an ideal proton gas at 1 bar and 298.15 K. Using water as an anchoring solvent enables the referencing of the measured pH_abs_ values to the conventional aqueous pH scale, yielding ${\text{pH}}_{\text{abs}}^{{\text{H}}_{2}\text{O}}$ values. Hence, metrological traceability can be achieved through the use of commonly available dilute aqueous buffer CRMs. The use of this scale has been demonstrated for solvent mixtures typically used in: liquid chromatography [33], aqueous buffers [34], water-ethanol mixtures [[34], [35], [36]], water-methanol and water-acetonitrile mixtures [35], as well as TRIS-buffered artificial seawater (ionic strength ∼ 0.7 mol kg^−1^) [36]. ${\text{pH}}_{\text{abs}}^{{\text{H}}_{2}\text{O}}$ measurements rely on the use of a salt bridge free of solvent, such as an ionic liquid (IL), instead of the more common aqueous KCl salt bridge. The ionic liquid salt bridge (ILSB) offers advantages in terms of the cancelation of LJPs between each solution under test and the IL and, therefore, contributes to increasing the reliability of measurements made in non-aqueous mixtures. Studies on solutions composed of different solvents, including ethanol [[37], [38], [39]], have shown that the IL triethylpentylammonium bis(trifluoromethanesulfonyl)imide, [N_2225_][NTf_2_], eliminates the LJPs within a consistency standard deviation corresponding to pH ∼ 0.1, this is equivalent to the uncertainty (coverage factor, *k* = 1) assigned to the residual liquid junction potential (RLJP) [21] of 7 mV.

In the present paper, several methods of measuring the acidity of water-ethanol mixtures are demonstrated and their strengths and weaknesses evaluated. These methods include: (1) the use of a commercial combination pH electrodes (aqueous KCl-based filling solution) in combination with a commercial pH meter, (2) differential potentiometry (reference method) for ${\text{pH}}_{\text{abs}}^{{\text{H}}_{2}\text{O}}$ measurements using a specialized (solid-contact) glass electrode half-cell and an ILSB [40], and (3) a new combination between a glass electrode (GE) half-cell and a Ag/AgCl reference electrode with an IL filling solution.

## 2 Materials and methods

### 2.1 Reagents and solutions preparation

Dilute, aqueous buffers were purchased from Hach Lange (with traceability to DFM primary (aqueous) pH buffers): phthalate, phosphate, and borate buffers, with assigned pH values (at 25 °C) of 4.005 (S11M002), 6.865 (S11M003), 7.00 (S11M004), and 9.18 (S11M006). Standard uncertainties assigned by the manufacturer are 0.005 pH at 25 °C.

Ionic liquid (C_13_H_26_F_6_N_2_O_4_S_2_, [N_2225_][NTf_2_]) was obtained from Iolitec GmbH (Heilbornn, Germany; courtesy of Dr V. Radtke, University of Freiburg).

Anhydrous ethanol was purchased from VWR (99.97% purity), the ethanol was used as purchased without any further treatment. The water present in the purchased ethanol (as stated by the manufacturer) was not taken into account; the anhydrous ethanol was treated as ‘pure ethanol’. Ultra-pure water (UPW) was obtained from a Milli-Q system (Millipore, Merck), and used without any further treatment, i.e., without purging of CO_2_. Used buffer salts were as follows: potassium hydrogen phthalate (KHC_8_H_8_O_4_, 99.97%, VWR), disodium hydrogen orthophosphate (Na_2_HPO_4_, 99.0%, VWR), potassium hydrogen orthophosphate (KH_2_PO_4_, 99.5%, VWR), and sodium tetraborate decahydrate (Na_2_B_4_O_7_◦10H_2_O, 101.8%, VWR). Prepared buffered solutions are referred to as: phthalate (KHC_8_H_8_O_4_), phosphate (1:1 Na_2_HPO_4_: KH_2_PO_4_), and borate (Na_2_B_4_O_7_◦10H_2_O).

The water-ethanol mixture was made on a wt% basis: 50-50 wt% UPW-anhydrous ethanol. Each buffer was prepared as: phthalate (0.05 mol kg^−1^), phosphate (0.013 m Na_2_HPO_4_: 0.015 m KH_2_PO_4_), and borate (0.01 mol kg^−1^). Buffered water-ethanol mixtures were made by first dissolving the required mass of (un-dried) salt into the mass of water, followed by the addition of ethanol. Issues were encountered keeping the borate salt in solution, so fresh solutions were manufactured each week according to the same formulation (0.01 mol kg^−1^ borate). Buffered and un-buffered water-ethanol mixtures were tightly sealed in 500 mL blue-cap borosilicate bottles, and stored under dark, ventilated conditions (23.0 °C ± 0.5 °C, 45% ± 5% relative humidity) until immediately before use.

### 2.2 Measurement procedures

#### 2.2.1 Commercial combination pH electrode

A commercial combination pH electrode (238160 Hamilton Single-Pore) with Skylyte inner filling solution (likely aqueous based), known to be reliable in dilute, aqueous buffer CRMs [41] was employed.

The electrochemical cell can be written:

Glass electrode | analyzed solution || Skylyte | AgCl (s)-Ag Cell (I)

Where || represents the single-pore geometry of the liquid junction in this particular type of commercial combination glass electrode.

Two sets of experiments were carried out using aqueous buffer CRMs, buffers prepared in a water-ethanol mixture (50 wt%), and an un-buffered solution. A different notation for the experimental pH values obtained using glass combination electrode will be used in this paper to distinguish between the values obtained after calibration with aqueous (pH) and water-alcohol (${\text{pH}}^{\text{*}}$) matrices.

Experiment 1 The water-ethanol solutions were treated as unknown samples and their pH was determined at 25.0 °C with the combined pH electrode after three-point calibration with classical aqueous buffer solutions of low ionic strength (nominal pH 4.005, 7.000, and 9.180).

pH(X) of an unknown solution was then obtained from Eq. (1):

(1) $$\text{pH}\left(\text{X}\right)=\frac{\text{E}\left(\text{x}\right)-{\text{E}}^{\text{′}}}{\text{k′}}$$

where *E(X)* is the measured potential difference, k' and *E′* are, respectively, the practical slope (mV·pH^−1^) and the intercept (mV) obtained after calibration of the glass electrode with aqueous buffers.

This experiment is representative of the current practice in routine laboratory measurements [24]. Solutions were measured on two different days in the sequence: aqueous CRMs, buffered water-ethanol mixtures, un-buffered water-ethanol. For same-day measurements, aqueous pH 4.005 and 9.18 measurements were repeated before and after measurement of water-ethanol solutions. These potential readings were found to be identical within 0.1 mV (the resolution of the pH meter) for both aqueous CRMs (same-day measurements).

Experiment 2 Two of the buffers prepared in water-ethanol were used to calibrate the combination glass electrodes setup, whereas the third buffer, as well as the un-buffered water-ethanol solution, were treated as unknown samples.

In this case ${\text{pH}}^{\text{*}}\left(\text{X}\right)$ of an unknown solution is obtained from the equation below:

(2) $${\text{pH}}^{\text{*}}\left(\text{X}\right)=\frac{\text{E}\left(\text{x}\right)-{\text{E}}^{\text{″}}}{\text{k″}}$$

where k’’ and *E″* are, respectively, the practical slope (mV·pH*^−1^) and the intercept (mV) obtained after calibration of the glass electrode with water-ethanol buffers.

Calibration parameters, i.e., slope (mV·pH^−1^) and intercept (mV), were calculated manually by plotting the measured potential as a function of the standard pH values. Electrode parameters, and their uncertainties, were calculated by least square minimization employing in-house software (DFM_LSQ). Same-day potential measurements and calibration curves were employed to calculated pH and pH* values.

Each analyzed solution was transferred to a ∼100 mL borosilicate glass vessel, a magnetic stir bar was inserted, and the glass vessel placed into a thermostating water bath equipped with magnetic stirring (Isotech Hyperion 4936). Stirring was kept at a constant rate for all solutions. A calibrated Pt100 resistance-type thermometer (AM1760-9 Accumac Technology Inc., USA) was inserted into the stirring solution such that thermometer and pH probe were inserted to the same depth. Temperature was displayed on a digital temperature readout (Fluke 1502A), and recorded along with each potential measurement. The pH electrode was connected to a pH meter (Orion VersaStar Pro, Thermofisher) set in ‘potential’ mode. The potential characteristics of this pH meter were previously verified against a calibrated source between - 400 mV and +400 mV. Additionally, the uncertainty contributions to the potential measurement for the combination of pH electrode and pH meter were assessed for uncertainty arising from calibration, repeatability, and reproducibility in dilute, aqueous buffers (0.6 mV). This methodology of measuring pH and pH* is similar to that described in EN15490 [7], although the filling solutions of the pH electrodes differ, with 1 mol L^−1^ LiCl in 99 vol% ethanol being specified in the EN standard.

Thermometer and pH electrode were inserted into each new solution, and the temperature was allowed to stabilize until the thermometer read 25.00 °C ± 0.03 °C. The temperature coefficients of the aqueous CRMs (calculated from data provided by the manufacturer) reveal that ± 0.03 °C has no significant influence on the pH value. The temperature coefficients of buffered water-ethanol solutions is similar to the aqueous CRMs, the higher value, of 0.01 pH·C°^−1^, being calculated for borate buffer. Therefore, the deviation in setpoint temperature is not expected to be a significant factor in the calculation of uncertainty, and so was omitted. Temperature equilibration took ∼20 min, after which a temperature and potential reading were taken.

#### 2.2.2 Direct differential potentiometry in 1 step (Reference method)

The reference method for ${\text{pH}}_{\text{abs}}^{{\text{H}}_{2}\text{O}}$ measurement is described in Ref. [34]. The cell employed consists of a thermostating water jacket, in which water is circulated at 25.0 °C (89203-000 VWR water circulator), and two measurement pots, which are connected by a ∼1 mm diameter capillary. The capillary is carefully filled with ionic liquid such that no air bubbles are present. For each pair of solutions to be measured, the two measurement pots are filled simultaneously with equal masses of the two solutions.

The electrochemical cell can be written:

Glass electrode |Solution 1|[N_2225_][NTf_2_]|Solution 2| Glass electrode Cell (II)

Where Solutions 1 and 2 are the aqueous pH buffers (pH 4.005 and 7.000 at 25 °C) and water-ethanol solutions under test respectively.The glass electrodes are a pair of solid contact glass (half-cell) electrodes (SCGEs) from the same production batch. SCGEs (EST-0601) from Izmeritelnaya Tekhnika [42] were calibrated with aqueous pH buffers against a calibrated RE (Radiometer Analytical REF201 red rod reference electrode, Hach Lange) consisting of a Ag/AgCl reference with saturated KCl (aq) filling solution. Their parameters, i.e., slope (mV pH^−1^) and intercept (mV) were determined without thermostating, at room temperature (∼22.5 °C). SCGEs are fitted into loose fitting lids and placed into each measurement pot. The potential difference in Cell (II) was recorded by a high input-impedance analyser (IM6eX, Zahner-Elektrik GmbH & CoKG, Germany) for 1 h, with a data point taken every 10 s. Solutions measured include two aqueous CRMs, and buffered water-ethanol mixtures.

For a measured potential difference, $\Delta E_{meas}$, between two solutions (Solutions 1 and 2), made using Electrodes 1 and 2, the $\Delta {\text{pH}}_{\text{abs}}^{{\text{H}}_{2}\text{O}}$ values were calculated from:

(3) $$\Delta {\text{pH}}_{\text{abs}}^{{\text{H}}_{2}\text{O}}=2\frac{\Delta {\text{E}}_{\text{meas}}+{\text{I}}_{1}-{\text{I}}_{2}-\text{LJP}}{{\text{k}}_{1}+{\text{k}}_{2}}+\text{s}+\text{R}$$

where k_1_ and k_2_ are, respectively, the slopes (mV pH^−1^), I_1_ and I_2_ are the intercepts (mV) of Electrodes 1 and 2, respectively; s is the consistency standard deviation of the ‘pH ladder’ minimization [33,34], and R is the reproducibility of measurement. Both s and R are assigned as zero with a standard uncertainty of ${\text{pH}}_{\text{abs}}^{{\text{H}}_{2}\text{O}}$ = 0.01.

The ILSB is soluble in ethanol, and so the cell is completely cleaned and the IL replaced after water-ethanol has been measured in one of the measurement pots. Based on the measured potential differences, and the assigned (conventional) pH values of the aqueous CRMs, the ${\text{pH}}_{\text{abs}}^{{\text{H}}_{2}\text{O}}$ values are calculated using the ‘pH ladder’ method [33]. The uncertainty of the potential measurements is taken to be the standard deviation over the final 30 min interval (1800 s–3600 s), which takes into account the drift observed during measurements. The uncertainty contribution of the LJP is taken to be 7 mV [39]. This is similar to that used by Ref. [43] (6.3 mV) for the same ILSB in contact with various water-ethanol solutions.

#### 2.2.3 Differential potentiometry in 2 steps: Glass (half-cell) electrode and Ag/AgCl (IL filling solution)

The cell can be written:

GE|analyzed solution|[N_2225_][NTf_2_]|3 M KCl (aq)|AgCl (s)-Ag Cell (III)

Where GE is the glass (half-cell) electrode (either SCGE or commercial GE), and the RE consists of a Ag/AgCl reference with two filling solutions: a 3 M KCl (aq) inner solution, and ionic liquid outer filling solution, acting as an salt bridge (ionic liquid salt bridge = ILSB). When equipped with the SCGE, this cell is designated Cell (III.1), and when equipped with the commercial GE, it is designated Cell (III.2).

The potential difference was recorded between a SCGE (half-cell) and a two-chambered Ag/AgCl reference electrode (6.0729.100 Metrohm) by the high input-impedance analyser (Zahner) for 10 min, or 30 min, with a data point taken every 10 s. The inner filling solution of the reference electrode was 3 M KCl (aq), and the outer filling solution was the IL, [N_2225_][NTf_2_]. The bottom stopper of the electrode was opened sufficiently to allow the IL to come into contact with the solution under test. This use of an IL filling solution is similar to that reported by Ref. [44] for aqueous solutions (conventional pH scale). Solutions measured include three aqueous CRMs, buffered and un-buffered water-ethanol mixtures. Solutions were placed in a beaker; GE and RE were immersed in the quiescent solution. Measurements were started immediately. The beaker was not thermostated, and solutions were likely at ambient temperature (∼23 °C–24 °C). While the temperature difference is expected to influence the measured ${\text{pH}}_{\text{abs}}^{{\text{H}}_{2}\text{O}}$ values to a small degree, a near identical method (without thermostating) was employed during a recent inter-laboratory comparison [36]. Results acquired during this comparison fit well with measurements using both the reference method (thermostated at 25 °C, Cell (II)), and data from other laboratories (E_N_ < 1 [45]).

${\text{pH}}_{\text{abs}}^{{\text{H}}_{2}\text{O}}$ values were calculated from:

(4) $${\text{pH}}_{\text{abs}}^{{\text{H}}_{2}\text{O}}=\frac{\Delta {\text{E}}_{\text{meas}}-\text{I}}{\text{k}}$$

where *ΔE*_*meas*_ is the measured potential of Cell (III), I and k represent the intercept and slope, respectively, of the GE, determined based on an initial calibration step (same day measurements). For aqueous pH buffers, data was acquired for 10 min; for buffered and un-buffered water-ethanol solutions, data was acquired for 30 min.

The value and uncertainty of the slope and intercept were calculated using the DFM_LSQ software, using the data acquired for the aqueous pH buffers. The uncertainty of the potential measurement is taken as the standard deviation of data acquired over a 10 min period, between t = 1200 s and t = 1800 s, taking into account the drift observed during measurement. Additionally, the dominant contribution to uncertainty remains the LJP (7 mV) [39] arising between the water-ethanol solution and the ILSB, and so the difference in temperature of measurement (vs. 25 °C) has been neglected.

${\text{pH}}_{\text{abs}}^{{\text{H}}_{2}\text{O}}$ values were also measured in an identical manner using a commercial GE (E11M001, Radiometer Medical Aps, Denmark) and the Ag/AgCl RE (Metrohm) with 3 M KCl inner filling solution and [N_2225_][NTf_2_] IL outer filling solution. This arrangement is designated Cell (III.2).

## 3 Results and discussion

### 3.1 Commercial combination pH electrode

Molality of phthalate (0.05 m) and borate (0.01 m) were adopted from pH standards in aqueous solution [20]. However, due to solubility problems, the molality of phosphate buffers was decreased to 0.015 m. The pH values of buffered water-ethanol mixtures obtained with the primary system, and considering the mixture as continuous (single phase) solvents, are denoted pH*_p_, with the subscript p as a symbol for ‘primary method’ for the solvent-specific scale (50-50 wt% water-ethanol). These buffered water-ethanol solutions were not certified using the primary method but were assumed to have identical pH*_p_ (not conventional aqueous pH) values to those measured at LNE using the primary method (Table 1), carried out as described in Ref. [31].

**Table 1:** Primary-method assigned pH values for buffered 50-50 wt% water-ethanol mixtures (measured at LNE).

Buffer salt	Molality (mol·kg^−1^)	Primary pH^b^_p_	Reference	
Phthalate	0.01	5.18	CCQM-P152^a^ [31];	
1:1 phosphate	0.015:0.015	7.75	b	
Borate	0.05	10.74	–	

a Demonstrated during Consultative Committee for Metrology in Chemistry and Biology (CCQM) Pilot Comparison (CCQM-P152), un-published data.

b Value comparable with other national metrology institutes (NMIs) (Germany, Brazil, and Japan).

The focus of this present work is not the measurement of pH*_p_ by the primary method (Table 1), so these values are employed at 25 °C without further discussion. Within the European Metrology Research Program (EMRP) Joint Research Project (JRP) Biofuels [46] an inter-laboratory comparison was organized (data not published) to validate the primary method used to assign pH*_p_ values for buffer solutions prepared in the 50-50 wt% water-ethanol mixture. The exercise showed that following the method described in the experimental section, it is possible to prepare pH buffer solutions within an expanded uncertainty (*k* = 2) of 0.01 pH*. Therefore, all buffered water-ethanol mixtures were assigned a pH* standard uncertainty (*k* = 1) of 0.005 at 25 °C.

Due to the nature of the GE [47], the calibration of the pH electrode was repeated each day.

The primary-method traceable, manufacturer assigned, pH values of the aqueous pH buffers were used to calibrate the combination glass electrode, giving slopes of −58.97(0.18) mV·pH^−1^ and -58.99(0.18) mV·pH^−1^ on days 1 and 2, respectively. The practical slopes are very close to the ideal values at 25 °C indicating the expected sub-Nernstian behavior of electrodes and their adequacy for the measurements [47]. Table 2 shows the measured potentials for the three aqueous CRMs, buffered and un-buffered water-ethanol mixtures.

**Table 2:** Potentials measured for buffered and un-buffered aqueous and water-ethanol mixtures on two separate days, using a commercial combination pH electrode and pH meter.

Solvent	Buffer	Potential (mV)	
		Day 1	Day 2
Aqueous	Phthalate	184.7	184.9
	Phosphate (pH 7)	8.5	8.6
	Borate	−120.5	−120.4
50-50 wt% water-ethanol	Phthalate	103.6	104.9
	Phosphate	−54.2	−51.8
	Borate	−218.0	−222.6
	Un-buffered	9.2	−4.6

As shown in Table 2, the measured potentials for the three aqueous CRMs vary slightly, but within standard uncertainty (0.005 pH), between days 1 and 2.

Similarly, the primary-method assigned pH* values of 50-50 wt% water-ethanol buffered solutions were used to calibrate the same electrode. This gave slopes of −57.84 (0.17) mV·pH*^−1^ and −58.90(0.17) mV·pH*^−1^on day 1 and 2, respectively. These values are similar to those obtained using aqueous pH buffer calibration on the conventional pH scale (i.e., mV·pH^−1^), as expected for combination glass electrodes.

For each buffered solution, the pH values calculated on two separate measurement days are in agreement, within expanded uncertainty (Table 3), and therefore E_N_ < 1 [45]. It is worth emphasizing that for this experimental configuration, the value and uncertainty contribution of the LJP between water and water-ethanol mixture were not included in the calculation of final pH values and uncertainties for water-ethanol solutions. Due to the magnitude of the LJP being unknown, though likely to be considerable [21,37], the true pH uncertainties are very likely to be larger than reported here. The unknown contribution of the LJP has previously been stressed as one of the issues with the ASTM D6423-14 methodology for (anhydrous) ethanol, and the metrological traceability of pHe measurements [17].

**Table 3:** pH and pH* values calculated at 25 °C for buffered and un-buffered water-ethanol solutions. For each measurement day, data for calibration curve(s) was acquired. The indicated standard uncertainties (*k* = 1) do not include any contributions from LJP.

Buffer	Assigned pH*_p_	Day 1		Day 2	
		pH^a^	pH*^b^	pH^a^	pH*^b^
Phthalate	5.18	5.33 ± 0.03	Used for calibration	5.36 ± 0.03	Used for calibration
Phosphate	7.75	8.06 ± 0.04	7.91 ± 0.04	8.02 ± 0.03	7.84 ± 0.04
Borate	10.74	10.84 ± 0.04	Used for calibration	10.92 ± 0.04	Used for calibration
Un-buffered	–	6.98 ± 0.04	6.81 ± 0.04	7.22 ± 0.04	7.04 ± 0.03

a Calculated using Eq. (1).

b Calculated using Eq. (2).

The obtained coefficients from the electrode calibration were used to calculate the pH values for the water-ethanol mixtures, measured on the same day. The agreement between measurement days indicates that any changes occurring to the GE as a results of contact with the ethanol [24] were reversible, at least in the short term. The assigned uncertainty for calculated pH values takes into consideration only contributions coming from the measured potential, slope, and intercept. The overall uncertainty was larger in the case of un-buffered water-ethanol, as the displayed potential values showed a 1 mV–2 mV variability over a short time scale (reading fluctuation). This may be due to an unstable ion equilibrium at the glass electrode due to the low ionic strength of the un-buffered water-ethanol mixture.

As expected, for all buffered water-ethanol solutions, pH values (Eq. (1)) are consistently not in agreement with primary-method assigned pH values, pH*_p_ (Table 3). The differences, ranging from +0.1 pH units for borate to +0.3 pH units for phosphate buffer, may be attributable to unknown changes in sign and magnitude of the LJPs established between the calibrants (aqueous) and the solution under test (50-50 wt% water-ethanol), each versus the inner filling solution (likely aqueous based) of the combination pH electrode [21].

Such values can be considered as corrections to be applied to pH values furnished by a pH electrode calibrated with aqueous buffer solutions. Hence, pH* values can be obtained by subtracting a factor δ as shown in equation below:

(5) $$pH^{*}=pH-\delta$$

However, the correction is expected to be a function of the solvent composition only [23] and should not depend on the nature of the buffering solutes in the solutions. Therefore, as all the analyzed buffers were prepared in the same solvent, a single correction specific for 50-50 wt% water-ethanol mixtures has been calculated as the mean of the individual corrections obtained for the 3 buffers measured over 2 different days. The value reported from measurements carried out in the present paper is δ = 0.20, with a standard deviation of 0.03. This value fits very well with other values reported in the literature for the same mixture, and tabulated in Refs. [48,49], i.e., 0.17, 0.21, and 0.29.

The calculated pH* values for the phosphate buffer (Eq. (2)), are also not in agreement with those assigned using the primary method (pH*_P_ = 7.75 at 25 °C), but are closer than calculated using the aqueous buffers as calibrants (Eq. (1), Table 3). Differences between primary and secondary (using a glass (half-cell) electrode vs. RE) pH* values have previously been observed by Ref. [28]. Additionally, primary pH*_p_ values (LNE) are given for 0.015 m: 0.015 m phosphate, while the solution employed in this work was 0.013 m Na_2_HPO_4_: 0.015 m KH_2_PO_4_. This difference arises due to difficulties in weighing out small quantities of un-dried Na_2_HPO_4_ salt (salt clumping) and might also partially explain the observed bias between pH*_p_ and pH*.

The calculated pH and pH* values for the un-buffered water-ethanol mixture shown in Table 3 are not in agreement between measurements performed on separate measurement days. This arises as the un-buffered solution is not stable with time, even in a sealed container. This conclusion is supported by the high drift and lack of repeatability reported by Heering et al. [43]. However, the values obtained for this solution confirm the applicability of a correction of δ = 0.20 (Eq. (5)).

The pH of un-buffered, and additive-free, approximately 50-50 wt% water-ethanol has previously been measured using aqueous buffer calibrated commercial pH electrodes [49]. In that work, solutions consisting of 51.6 vol% to 53.20 vol% ethanol (∼50-50 wt%, assuming 0.789 g mL^−1^ density of ethanol) were prepared with distilled water, with pH values between 5.56 and 6.97 at room temperature being reported. Tabulated literature values, reported therein, includes a 55.9 vol% ethanol solution with an assigned pH of 7.44. The pH values of un-buffered water-ethanol measured in this work (Table 3) fall within the range of previously reported values.

For all analyzed water-ethanol solutions, it was observed that both pH and pH* values are not stable with time. Changes in solvent composition might occur between the 2 days of measurement. From vapor-liquid equilibrium data of water-ethanol mixtures, the vapor in equilibrium with a solution contains around a 0.55 mol fraction of ethanol, is about 75% of ethanol [50]. This vapor could be lost when the storage bottle is opened.

Additionally, it does not appear that un-buffered water-ethanol mixtures can be manufactured reproducibly. A number of factors can also change the pH or pH* of this un-buffered mixture:

- on what basis the water:ethanol ratio is determined (e.g., volume %, weight %, masses corrected for air buoyancy, etc.),
- initial quality of the ethanol (i.e., presence of water and other contaminants),
- treatment and quality of the water employed,
- bottles used (size, material, method of sealing) for storage,
- environmental conditions and duration of storage.

These differences complicate the issue of assigning a ‘correct’ pH* (50-50 wt% water-ethanol solvent-specific scale) value to un-buffered water-ethanol. The results presented here suggest the value lies between 6.75 and 7.04 at 25 °C.

### 3.2 Direct differential potentiometry for ${\text{pH}}_{\text{abs}}^{{\text{H}}_{2}\text{O}}$ measurements (reference method)

**Fig. 1:**
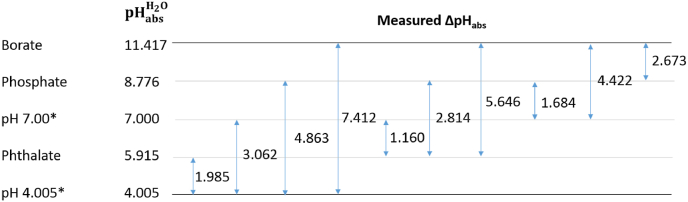
pH ladder and calculated ${\text{pH}}_{\text{abs}}^{{\text{H}}_{2}\text{O}}$ values, where aqueous pH buffers (pH 4.005 and 7.000, 25 °C), indicated by *, were used as anchoring buffers.

The ${\text{pH}}_{\text{abs}}^{{\text{H}}_{2}\text{O}}$ values were calculated using the “pH ladder” method [33,34]. The constructed ladder is shown in Fig. 1, along with ${\text{pH}}_{\text{abs}}^{{\text{H}}_{2}\text{O}}$ values for the: 0.05 m phthalate, 0.013 m: 0.015 m phosphate, and 0.01 m borate water-ethanol mixtures.

**Fig. 2:**
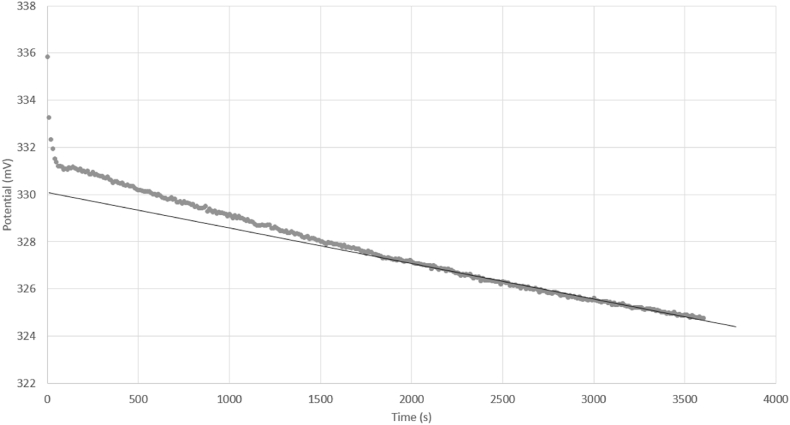
Potential difference measured using Cell (II), where the two solutions under test are phthalate and borate buffered water-ethanol. Solid line shows a linear fit of the last 30 min of data extrapolated to t = 0, where the slope of this line gives the drift rate (5.3 mV·h^−1^).

Measured potential differences were seen to drift over the course of the 1 h measurement (e.g., 5.3 mV·h^−1^, Fig. 2). This may be due to a combination of factors, including changes in solution composition (e.g., organic content) and gradual mixing of the ILSB into the water-ethanol solvent. Dissolution of the ILSB leads to an unstable junction between the two compartments, which further influences the stability of the signal. For this reason, the potential measured between the two glass electrodes was averaged over the same 30 min interval (t = 1800 s–3600 s) [35], and the uncertainty of the potential difference was assigned as the standard deviation, thus taking into account the observed drift.

The dominant contribution to the uncertainty of calculated ${\text{pH}}_{\text{abs}}^{{\text{H}}_{2}\text{O}}$ values is that assigned to the LJPs formed between the IL and the solutions in each compartment of the differential potentiometric cell. The inclusion of this uncertainty contribution is essential for making the calculated ${\text{pH}}_{\text{abs}}^{{\text{H}}_{2}\text{O}}$ values traceable to the current definition of pH (the conventional aqueous pH scale [20]). The overall standard uncertainty (*k* = 1) of assigned ${\text{pH}}_{\text{abs}}^{{\text{H}}_{2}\text{O}}$ values with this method is 0.13. The traceability of ${\text{pH}}_{\text{abs}}^{{\text{H}}_{2}\text{O}}$ values to the conventional pH scale represents one of the main advantages of its measurement by differential potentiometry. This is in contrast to (secondary) pH* measurements, which are traceable to pH*_P_ reference materials, which are largely unavailable for many water-solvent mixtures.

The added benefits of differential potentiometry measurements includes their ease of use compared to Harned cell (primary method) pH*_P_ determinations, including: shorter experiment time, ease of setup and clean-up, far fewer instrumental requirements (ability to use more readily available, and lower cost equipment), lower volume of solution consumed, elimination of the need for a convention as is required for conventional pH, i.e., the Bates-Guggenheim convention, and ease of application to multiple solvents and mixing ratios. Compared to ASTM D6423-14 [6] requirements, this method (Cell (II) configuration) allows the use of other commercially available glass (half-cell) electrodes [35,43], and data acquisition time [17] is not such a strict requirement. ASTM D6423-14 [6] requires the use of the Ross Orion SureFlow (Thermofisher) electrode for pHe measurements, and a reading should be taken 30 s following immersion of the probe into the water-ethanol mixture. The potential difference measured using the method described here (Cell (II) configuration) should be made within a few hours of bringing the water-ethanol solution into contract with the ILSB [43].

The calculated ${\text{pH}}_{\text{abs}}^{{\text{H}}_{2}\text{O}}$ values are numerically dissimilar to the primary-method assigned pH*_P_ (Table 1) and pH measured using a combination pH electrode (Cell (I)) (Table 3). Attempts were made to convert the pH*_P_ scale values to the ${\text{pH}}_{\text{abs}}^{{\text{H}}_{2}\text{O}}$ scale using the formula:

(6) $${\text{pH}}_{\text{abs}}^{{\text{H}}_{2}\text{O}}=-\frac{\Delta_{\text{tr}}{\text{G}}^{\text{o}}\left({\text{H}}^{+},{\text{ H}}_{2}\text{O}\to {\text{S}}_{2}\right)}{\text{RT}\ln\;10}+{\text{pH}}_{\text{P}}^{\text{*}}$$

where $\Delta_{\text{tr}}{\text{G}}^{\text{o}}\left({\text{H}}^{+},{\text{ H}}_{2}\text{O}\to {\text{S}}_{2}\right)$ is the Gibbs free energy of transfer of protons from water to the solvent (50-50 wt% water-ethanol), which is taken as −0.6 kcal mol^−1^ according to Ref. [51]. R is the ideal gas constant, and T is the temperature (in Kelvin, 298.15 K). For buffered water-ethanol solutions, the calculated ${\text{pH}}_{\text{abs}}^{{\text{H}}_{2}\text{O}}$ values are given in Table 4. The value of $\Delta_{\text{tr}}{\text{G}}^{\text{o}}\left({\text{H}}^{+},{\text{ H}}_{2}\text{O}\to {\text{S}}_{2}\right)$ used to calculate theoretical ${\text{pH}}_{\text{abs}}^{{\text{H}}_{2}\text{O}}$ represents the average of the computed results obtained using four different models [51]. The dispersion of 0.12 kcal mol^−1^ within the ${\text{Δ}}_{\text{tr}}{\text{G}}^{\text{o}}\left({\text{H}}^{+},{\text{ H}}_{2}\text{O}\to {\text{S}}_{2}\right)$ value translates into a standard uncertainty of approximately 0.1 pH for theoretical ${\text{pH}}_{\text{abs}}^{{\text{H}}_{2}\text{O}}$ values. Thus, the uncertainty of the theoretical value is of the same order of magnitude as the uncertainty of 0.12–0.13. ${\text{pH}}_{\text{abs}}^{{\text{H}}_{2}\text{O}}$ estimated for the experimental values. For phthalate and borate buffers there is no statistical difference between the theoretical (Eq. (6)) and experimental ${\text{pH}}_{\text{abs}}^{{\text{H}}_{2}\text{O}}$ values, as E_N_ < 1 [45]. For phosphate buffer the values are dissimilar and confirm the potential impact of problems with solution preparation highlighted during the measurements with a combination glass electrode.

### 3.3 Glass (half-cell) electrode and RE

Determined slope were −57.69(0.14) mV·pH^−1^ for SCGE and −58.75(0.10) mV·pH^−1^ for commercial GE in Cell (III.1) and Cell (III.2), respectively. The un-buffered mixture was analyzed with the SCGE for 3 consecutive days, with a fresh solution prepared each time. ${\text{pH}}_{\text{abs}}^{{\text{H}}_{2}\text{O}}$ values were calculated based on calibrations performed daily.

**Fig. 3:**
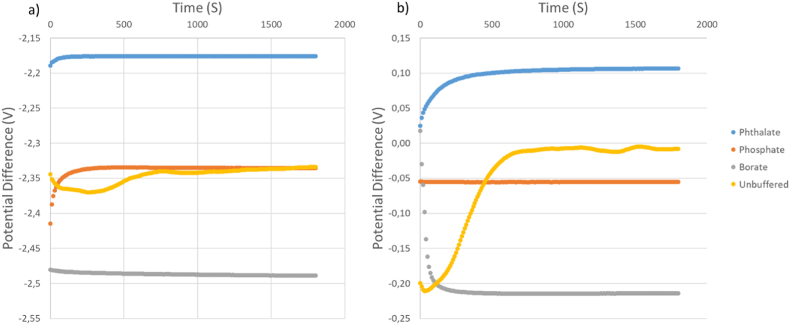
Measured potential difference for (a) SCGE vs. RE and (b) commercial GE vs. RE. Where RE (Ag/AgCl) has two filling solutions: 3 M KCl (aq) inner filling, and [N_2225_][NTf_2_] IL outer filling solution. Measures performed non-thermostated (23 °C ± 1 °C).

Fig. 3 shows the measured potentials against time for all the water-ethanol solutions using both configurations for Cell (III). A stable drift rate was observed between t = 1200 s and t = 1800 s (10 min of data) in each case. Signal instability observed at the beginning of measurement can vary between buffer samples and even between replicates of the same buffer. It is assumed that the observed instability is driven by temperature stabilization and the establishment of stable liquid junctions in the system. The potential differences are dissimilar between Cell (III) using SCGE and commercial electrodes, but gave identical ${\text{pH}}_{\text{abs}}^{{\text{H}}_{2}\text{O}}$ values. The overall standard uncertainty of assigned ${\text{pH}}_{\text{abs}}^{{\text{H}}_{2}\text{O}}$ values with this method is 0.12.

Table 4 summarizes the ${\text{pH}}_{\text{abs}}^{{\text{H}}_{2}\text{O}}$ values obtained using 2 methods: (1) differential method (reference method) based on SCGE vs. SCGE (Cell (II)), and (2) glass (half-cell) electrodes vs. RE with ILSB in 2 configurations: (a) SCGE (Cell (III.1)), and (b) a commercial GE (Cell (III.2)).

For all buffered solutions reported in Table 4, the determined ${\text{pH}}_{\text{abs}}^{{\text{H}}_{2}\text{O}}$ values are in agreement within uncertainty between both configurations of Cell (III): using a SCGE (Cell (III.1)) or a commercial GE (Cell (III.2)). This suggests that ${\text{pH}}_{\text{abs}}^{{\text{H}}_{2}\text{O}}$ measurements are not limited to the use of SCGEs [42] that are difficult to obtain, but can be performed using the Cell (III) configuration with commercially available GEs. This confirms the findings of [43] regarding the use of GE pairs in the Cell (II) configuration, for water-ethanol mixtures with ionic additives, specifically that commercial GE (half-cells) can be employed to the measure ${\text{pH}}_{\text{abs}}^{{\text{H}}_{2}\text{O}}$ of water-ethanol mixtures.

For each measurement day, a new solution of un-buffered water-ethanol was prepared from the same ethanol source, these did not show consistent values. This further emphasizes the influence of preparation ingredients and conditions on the ${\text{pH}}_{\text{abs}}^{{\text{H}}_{2}\text{O}}$ values of un-buffered solvents. A ${\text{pH}}_{\text{abs}}^{{\text{H}}_{2}\text{O}}$ value of 6.82–7.80 has previously been reported in Ref. [43] and 5.85 to 8.12 in Ref. [35] for 50-50 wt% water-ethanol mixtures without additives (buffers, or other salts) at 25 °C. The quality of the results reported therein [35,43] was explained by the differences between preparation ingredients and between electrodes employed in the Cell (II) configuration. These reported values are numerically similar to the ${\text{pH}}_{\text{abs}}^{{\text{H}}_{2}\text{O}}$ values reported in the present paper. The absence of a buffering agent from the water-ethanol mixture makes this solution much more sensitive to different aspects related to the solution preparation like sources and purities of the (anhydrous) ethanol, the quality of water employed (e.g., UPW (this work) vs. de-ionized water [43]), and initial pH of the water employed [49]. These could explain the high dispersion of ${\text{pH}}_{\text{abs}}^{{\text{H}}_{2}\text{O}}$ data obtained for the un-buffered water-ethanol mixture [35].

During a recent inter-laboratory comparison, a 0.015 m: 0.015 m phosphate 50-50 wt% water-ethanol mixture was prepared by collaborators of the UnipHied EMPIR research project [36]. The ${\text{pH}}_{\text{abs}}^{{\text{H}}_{2}\text{O}}$ results reported by DFM during this comparison are compared Table 5 with the data obtained from the present study. The consistency of ${\text{pH}}_{\text{abs}}^{{\text{H}}_{2}\text{O}}$ values between these 2 different studies demonstrate the robustness of the absolute pH concept and measurement methodologies embodied in cells (II) and (III).

**Table 4:** Theoretical (Eq. (6)) and experimental ${\text{pH}}_{\text{abs}}^{{\text{H}}_{2}\text{O}}$ values for 50-50 wt% water-ethanol solutions obtained with 3 methods: differential method (reference method, Cell (II)), SCGE vs. RE with ILSB (Cell (III.1)), and commercial GE vs. RE with ILSB (Cell (III.2). Standard uncertainty (k = 1) for the experimental values includes the contribution of the LJP.

Solution	Molality (mol kg^−1^)	Theoretical $pH_{abs}^{H_{2}O}$^b^	$pH_{abs}^{H_{2}O}$ with reference method (cell II)	$pH_{abs}^{H_{2}O}$ with SCGE vs RE ILSB (cell III.1)	$pH_{abs}^{H_{2}O}$ with commercial GE vs RE ILSB (cell III.2)
Phthalate	0.05	5.62 ± 0.1	5.92 ± 0.13	6.08 ± 0.12	6.03 ± 0.12
Phosphate	0.013: 0.015	8.19 ± 0.1	8.78 ± 0.13	8.85 ± 0.12	8.78 ± 0.12
Borate	0.01	11.18 ± 0.1	11.42 ± 0.13	11.49 ± 0.12	11.49 ± 0.12
Un-buffered^a^	/	/	/	7.52 ± 0.12 7.09 ± 0.12 8.89 ± 0.12	7.89 ± 0.12

a New un-buffered 50-50 wt% water-ethanol solution freshly prepared for each measurement.

b Calculated using Eq. (6).

**Table 5:** Comparison of ${\text{pH}}_{\text{abs}}^{{\text{H}}_{2}\text{O}}$ values obtained for equimolal (0.015 m) phosphate buffer prepared in water-ethanol mixture (50 wt%) between an interlaboratory comparison organized within Uniphied EMPIR project [36] and the present study. Values are given with their associated standard uncertainties (k = 1).

Method	Interlaboratory comparison^a^	This work
Cell (II)	8.73 ± 0.13 (DFM) 8.75 ± 0.13 (UT)^b^	8.78 ± 0.13
Cell (III.1)	8.67 ± 0.13	8.85 ± 0.13
Cell (III.2)	8.75 ± 0.13	8.78 ± 0.13

a [36].

b Assigned as reference value by University of Tartu.

The robustness of the concept is supported by additional data obtained using these three cell configurations (Table 5) for ammonium formate (10 mM) buffer in pure ethanol [36]. This suggests that the Cell (III) configuration gives valid measurements on the ${\text{pH}}_{\text{abs}}^{{\text{H}}_{2}\text{O}}$ scale, and can be more readily adopted by analytical laboratories.

## 4 Conclusions

Several methods for measuring the acidity of complex solutions were investigated through measurements with buffered and un-buffered water-ethanol mixtures (50 wt%). $pH_{abs}^{H_{2}O}$ measurement results obtained with cell II, and cell III, demonstrate the possibility of overcoming some practical (relating to liquid junction potentials) and theoretical (that the acidity scale is linked to the standard state and thus to the nature of the solvent) difficulties attributed to traditional pH measurements made with glass electrodes (represented by cell I).

The present study highlights the possibility of measuring $pH_{abs}^{H_{2}O}$ using conventional and easily manageable measurement equipment i.e. cell III. It consists of a glass electrode half-cell and a Ag/AgCl double junction reference electrode with two filling solutions: concentrated KCl (aq, min. 3 M) as the inner filling solution, and the ionic liquid [N2225][NTf2] as the outer filling solution, which acts as an ionic liquid salt bridge (ILSB). This configuration can readily be used in analytical laboratories to assign ${\text{pH}}_{\text{abs}}^{{\text{H}}_{2}\text{O}}$ ranges to bioethanol thus enabling these legislated measurements to be metrologically sound, as compared to the current pHe values. This is due to ${\text{pH}}_{\text{abs}}^{{\text{H}}_{2}\text{O}}$ measurement results being traceable to the current definition of (conventional) pH and comparable between different solvents.

Consistent ${\text{pH}}_{\text{abs}}^{{\text{H}}_{2}\text{O}}$ values were found for buffer solutions. However, these solutions are not expected to be stable long-term, and may only be useable within hours or days of first opening the sample bottles. These preliminary findings suggest against the pursuit of certifying these buffered water-ethanol mixtures as certified reference materials. Alternatively, precise recipes, including instructions for quality and treatment of salt(s), water, and ethanol, mixing, storage, and recommended bottle material (e.g., glass, plastic), could be made available. Buffered water-ethanol mixtures, with assigned ${\text{pH}}_{\text{abs}}^{{\text{H}}_{2}\text{O}}$ values (and associated uncertainty), could then be reproducibly created for use in-situ (i.e., at a routine measurement laboratory). This could be considered analogous to the existing IUPAC recommendations for molal electrolytic conductivity standards [52].

## CRediT authorship contribution statement

**L. Deleebeeck:** Conceptualization, Data curation, Formal analysis, Investigation, Methodology, Resources, Supervision, Visualization, Writing – original draft, Writing – review & editing. **A. Snedden:** Data curation, Funding acquisition, Formal analysis, Formal analysis, Methodology, Validation, Writing – review & editing. **D. Stoica:** Conceptualization, Data curation, Funding acquisition, Investigation, Methodology, Project administration, Visualization, Writing – review & editing.

## Declaration of competing interest

The authors declare that they have no known competing financial interests or personal relationships that could have appeared to influence the work reported in this paper.

## Glossary

pH: Defined as the negative logarithm of single free H+ activity according to internationally agreed IUPAC recommendation
pH*: pH value measured on specific solvent scale
pH*_P_: pH value on specific solvent scale assigned with the primary Harned cell
pH_abs_: absolute pH value expressing acidities in terms of chemical potentials
$pH_{abs}^{H_{2}O}$: : particular use of pHabs scale. The pHabsH2O-values in water will be equal to the conventional aqueous pH scale, and pHabsH2O-values in any other solvent/medium can be directly compared to the well-known aqueous pH scale
